# Cognitive enhancement through music therapy: meta-analytic evidence across clinical population

**DOI:** 10.3389/fpubh.2025.1735470

**Published:** 2026-01-08

**Authors:** Gabriella Jeanne Mulia, Yung-Hsiao Chiang, Stephanie Grace Maringka, John Chung-Che Wu, Hon-Ping Ma, Ju-Chi Ou, Kai-Yun Chen

**Affiliations:** 1 International Master Program in Medical Neuroscience, College of Medical Science and Technology, Taipei Medical University, Taipei, Taiwan; 2 Department of Surgery, School of Medicine, College of Medicine, Taipei Medical University, Taipei, Taiwan; 3 Department of Neurosurgery, Taipei Medical University Hospital, Taipei, Taiwan; 4 Research Center for Neuroscience, Taipei Medical University, Taipei, Taiwan; 5 Taipei Neuroscience Institute, Taipei Medical University, Taipei, Taiwan; 6 Department of Biomedicine, School of Life Sciences, Indonesia International Institute for Life Sciences (i3L), Jakarta, Indonesia; 7 Emergency Department, Shuang Ho Hospital, Taipei Medical University, Taipei, Taiwan; 8 Graduate Institute of Injury Prevention and Control, Taipei Medical University, Taipei, Taiwan; 9 PhD Program in Medical Neuroscience, Taipei Medical University, Taipei, Taiwan

**Keywords:** aging, cognitive enhancement, meta-analysis, MMSE, music therapy, neuroplasticity

## Abstract

**Introduction:**

Cognitive decline in aging populations presents a growing public health concern. Music therapy has emerged as a promising non-invasive approach to enhance cognitive function.

**Methods:**

This meta-analysis synthesized data from 14 studies (2010–2025) assessing Mini-Mental State Examination (MMSE) outcomes following music therapy interventions.

**Results:**

Results revealed a significant improvement in cognitive function (SMD = 0.46, 95% CI: 0.26 to 0.67), particularly with passive listening-based therapies administered for more than 3 months (SMD = 0.62, 95% CI: 0.05 to 1.20). Furthermore, non-RCT study designs and publications made after 2019 showed larger effects due to larger sample sizes and technological advancements. These findings support the hypothesis that auditory-based music therapy may facilitate neuroplasticity via modulation of the dopaminergic system and default mode network connectivity. The results underscore the therapeutic potential of music-based interventions as accessible, scalable, and non-invasive strategies for cognitive enhancement across diverse clinical populations.

**Discussion:**

Music therapy, especially passive listening interventions, shows significant promise for improving cognitive function in aging populations. Larger effects in recent non-RCT studies likely reflect technological advancements and improved methodologies. Future research should standardize protocols, examine long-term outcomes, and investigate the neurobiological mechanisms, particularly dopaminergic modulation and default mode network changes, to optimize therapeutic strategies and validate music therapy’s role in cognitive health.

**Systematic review registration:**

https://www.crd.york.ac.uk/PROSPERO/view/CRD420251004837, identifier PROSPERO (CRD420251004837).

## 1 Introduction

Cognitive impairment represents a pressing global health issue, particularly in aging populations, where the incidence of neurodegenerative conditions such as Alzheimer’s and Parkinson’s disease is rapidly increasing (1, 2). Cognitive function – encompassing memory, attention, executive control, and language – declines progressively with age and disease, significantly affecting quality of life, autonomy, and long-term health outcomes (3, 4). Traditional interventions for cognitive impairment have included pharmacological treatments and cognitive training; however, these approaches often show limited efficacy or present adverse side effects (5). This has prompted interest in non-invasive, scalable alternatives. Among these, auditory-based interventions, particularly music therapy, have shown promise for enhancing cognitive function via multisensory engagement and emotional stimulation (6, 7).

Music-based interventions may exert their effects through both behavioral and neurophysiological mechanisms. Functional neuroimaging studies have demonstrated that music activates widespread cortical and subcortical networks, including regions involved in memory, emotion, and executive control (8, 9). Moreover, auditory stimulation has been associated with modulation of dopaminergic transmission and enhanced connectivity within the default mode network (DMN), both of which are implicated in cognitive aging and neurodegeneration. Studies have found that activating the mesolimbic reward system which plays a central role in motivation, learning, and reward processing can modulate dopaminergic signaling, linking the emotional and pleasurable experience of music into memory and learning (10, 11). On the other side of this system is the DMN which comprises several brain structures, including the medial prefrontal cortex, anterior cingulate cortex, and posterior cingulate cortex, as well as the precuneus. The DMN is primarily responsible for directed thought and episodic memory, and its functional connectivity gradually declines through aging and in conditions such as Alzheimer’s disease. Emerging evidence from resting-state fMRI studies suggests that music therapy may enhance DMN connectivity and promote interactions between the DMN and task-positive networks to effectively support cognitive function improvements such as attention, memory, and executive function (12, 13). In clinical populations, especially people with early-stage dementia, trials have demonstrated that regular music therapy are associated with improvements in cognitive function and psychological symptoms, potentially through fortifying functional connectivity within and between networks such as the DMN and fronto-parietal systems (13). These findings suggest that music therapy may provide a non-pharmacological intervention to support cognition in aging and neurodegeneration.

Despite these findings, systematic reviews have yielded mixed results due to heterogeneous methodologies, intervention types, and outcome measures. For instance, a meta-analysis by Lin et al. (14) reported that music-based therapies showed improvements in the cognition of patients with dementia compared to conventional interventions measured with the Neuropsychiatric Inventory (NPI), whereas the meta-analysis by Bian et al. (15) reported similar findings using the Mini-Mental State Examination (MMSE) scale. The MMSE remains one of the most widely used tools for evaluating global cognitive function and intervention efficacy in clinical populations (16). Nevertheless, existing studies based on MMSE remain sparse and vary in terms of patient populations, intervention duration, and treatment parameters.

The present meta-analysis aims to quantify the effects of music therapy on MMSE scores. This study further investigates whether specific intervention modalities (e.g., passive listening vs. interactive therapies) and durations (e.g., short-term vs. long-term) are associated with differential cognitive outcomes to address the current gap and provide a reference for future clinical translation.

## 2 Methods

### 2.1 Search strategy and study selection

A systematic literature search was conducted in accordance with PRISMA guidelines and registered in the PROSPERO database (CRD420251004837). Searches were performed in PubMed, Embase, and Cochrane Library databases using combinations of Medical Subject Headings (MeSH) and keyword terms related to auditory-based interventions and cognitive outcomes. The search string included:

(“music therapy” OR “music-based intervention” OR “singing therapy”) AND (“Mini-Mental State Examination” OR “MMSE”).

Searches were limited to peer-reviewed articles published in English from 2010 to 2025. Eligible studies were required to be original full-text publications in peer-reviewed journals that included adult participants. Studies must have applied a music-based intervention and reported pre- and post-intervention Mini-Mental State Examination (MMSE) scores. Additionally, studies were required to employ a randomized controlled trial (RCT), non-randomized, or prospective clinical design. Studies were excluded if they were non-original publications such as reviews or protocols, did not use music based interventions, lacked MMSE data or sufficient outcome statistics, or focused on psychiatric or developmental disorders outside the scope of this analysis. The PICOS framework applied for this review was as follows:

P(Population): adults with clinical conditions other than psychiatric or developmental disorders.

I(Intervention): music-based interventions (listening, singing, movement).

C(Comparator): pre-intervention or non-music therapy control groups.

O(Outcomes): MMSE scores reported pre- and post-intervention.

### 2.2 Data extraction

Two independent reviewers (G. M., S. M.) extracted study data, including author, year, country, sample size, population characteristics, disease type, intervention method (listening, singing, movement), duration, and study design. Disagreements were resolved by a third reviewer (J. O.). Inter-rater agreement was quantified using Cohen’s kappa. Studies were further categorized into neurodegenerative or non-neurodegenerative conditions and grouped by treatment duration and modality.

### 2.3 Risk of bias assessment

Risk of bias for RCTs was assessed using the Cochrane Risk of Bias 2.0 (RoB-2) tool, applied using both intention-to-treat (ITT) and per-protocol (PP) approaches where applicable. Non-RCT (observational) studies were evaluated using the Newcastle-Ottawa Scale (NOS). Discrepancies in assessments were resolved by consensus among reviewers. Risk ratings were used to inform sensitivity analyses and evaluate study quality.

Funnel plot and sensitivity analysis were performed to evaluate publication bias and small-study effects, in addition to the robustness of study findings, respectively.

### 2.4 Outcome and factors

The primary clinical outcome measurements utilized in this analysis is the Mini-Mental State Examination (MMSE) used to measure cognitive function based on orientation to time and place, registration, attention and calculation, recall, and language. The measurement is done with a scoring system of 0–30 points, with higher scores indicating better function (17).

Several factors assessed in this meta-analysis include study design, disease or condition, treatments, duration, and publication year. These outcomes were selected to explore the potential sources of heterogeneity and assess the consistency of music therapy efficacy across the different factors.

### 2.5 Statistical analysis

All analyses were conducted using R (version 4.3.2, New York, United States) with the meta and metafor packages. Standardized mean differences (SMD) with 95% confidence intervals (CI) were computed for MMSE pre- and post-treatment scores using random-effects models. Heterogeneity was assessed via the I^2^ statistic. Egger’s test was performed to assess publication bias. A *p*-value of less than 0.05 indicates significant asymmetry in the funnel plot, suggesting the presence of publication bias. Subgroup analyses examined differences by study design (RCT vs. non-RCT), intervention modality (listening, singing, combination), duration (<7 days, 7 days–3 months, >3 months), disease category, and publication year (pre-2019 vs. post-2019). Studies that reported multiple follow-up assessments had each timepoint included into the corresponding duration subgroup. For instance, the study by Li et al. (18) included a follow-up both immediately post-treatment (<7 days) and 3 months post-treatment (7 days to 3 months). Statistical significance was defined as *p* < 0.05.

## 3 Results

**Figure 1:**
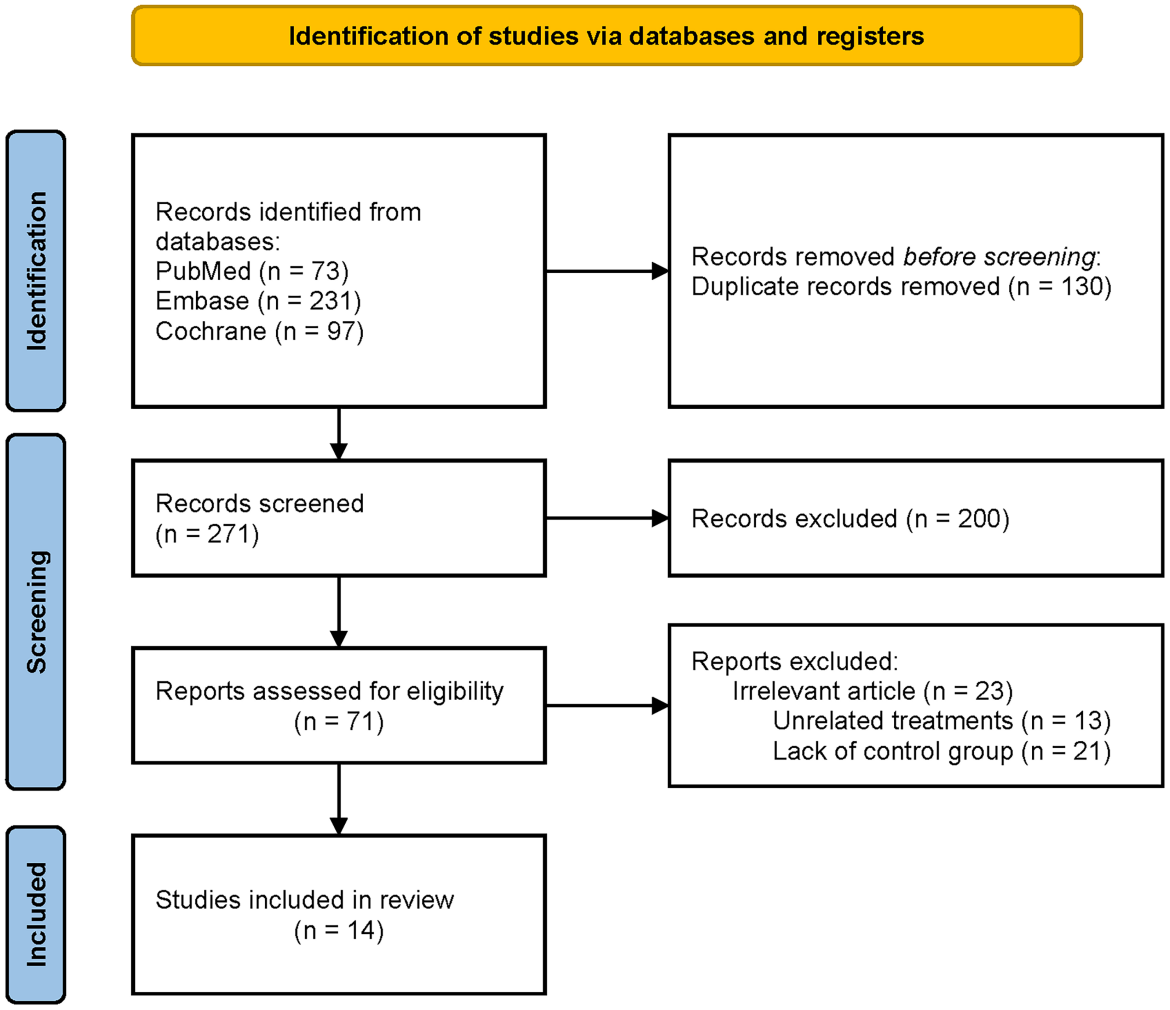
Diagram flow of the study selection.

The initial search yielded 401 records, with 130 duplicates removed. After title and abstract screening, 71 full-text articles were assessed for eligibility. Of these, 14 studies met the inclusion criteria and were included in the meta-analysis. The PRISMA flow chart summarizing the selection process is presented in Figure 1 (18–31).

Study characteristics for the 14 selected investigations are detailed in Table 1, encompassing author information, publication year, country of origin, study design, treatment conditions, clinical populations, participant demographics, and intervention protocols with associated durations. The included studies comprised both randomized controlled trials (RCTs) and non-randomized designs, with participants representing diverse clinical populations including orthopedic surgery patients (hip/knee procedures), individuals with mild cognitive impairment, and patients across the dementia spectrum from mild to severe stages. Sample sizes ranged considerably, with the largest cohort (*N* = 192) reported by Lyu et al. (26), demonstrating gender distributions of 40/57 (M/F) for the music intervention group and 39/57 for controls. Participant age ranges extended from 65 to over 85 years, with music-based interventions varying in modality (passive listening, active singing, instrumental performance, and movement-based activities) and temporal parameters (3 days to 6 months). Most studies showed participants had significant improvements in cognitive function, psychological symptoms, and quality of life; though studies by Li et al. (18), Pérez-Ros et al. (28), and Tang et al. (30) showed stable to no changes in cognitive function. However, the study by Li et al. (18) showed improvement in adjunct cognition, while another by Tang et al. (30) showed reduced apathy in participants.

**Table 1:** Summary of study characteristics.

First author	Country	Study design	Disease	Groups	Number of participants	Gender (M, F)	Age (mean ± SD)	Treatment	Duration	Principal results
Biasutti et al. (2018) (19)	Italy	RCT	Mild Cognitive Impairment	Music	18	4, 14	83.39 ± 7.81	Singing, playing	70 min/ biweekly/ 6 months	-Significant improvements across all tests, especially MMSE and clock drawing test – cognitive music training showed positive impact in the experimental group over time, with significant effects on executive functions, specifically in attention. -Effective for patients with mild to moderate cognitive impairment (MMSE ≤23).
				Control	17	8, 9	83.76 ± 6.16	Gymnastic activities	45 min/ biweekly/ 6 months	
Cetinkaya (2019) (20)	Turkey	RCT	Hip or knee surgery	Music	30	10,20	65–74y: 26 75–84y: 3 ≥85y: 1	Listening	20 min/ 3 times a day/ 3 postoperative days	-No significant difference in MMSE score, but significantly improved NEECHAM confusion scale score. -Listening to classical Turkish music improved cognitive function and reduced acute confusion in older adults undergoing hip and knee prosthesis surgery.
				Control	30	5,25	65–74y: 22 75–84y: 5 ≥85y: 3	Standard care	3 days	
Hong et al. (2011) (21)	South Korea	RCT	Mild Dementia	Music	15	2,28	78.3 ± 6.3	Listening, singing, playing	60 min/ once a week/ 16 weeks	-Songwriting music therapy program significantly improved MMSE-K score by 26%. -Songwriting-oriented music therapy significantly improved cognitive function – particularly in language function, orientation, and memory – of aged participants with dementia.
				Control	15			Standard care	16 weeks	
Kim (2020) (22)	South Korea	RCT	Mild Dementia	Music	18	3,15	80.6 ± 5.12	Listening, singing, playing	1 h/ 5 times a week/ 5 weeks (24 sessions)	-Recollection-based occupational therapy consisting of art, music, and horticultural activities significantly improved MMSE-K score in AD patients. -Intervention effectively improved cognitive function, reduced depression, and enhanced quality of life
				Control	17	6,11	77.88 ± 5.49	Standard care	5 weeks	
Kim et al. (2021) (23)	South Korea	RCT	Mild Cognitive Impairment	Music	20	6,14	80.6 ± 8.1	Listening, singing, movement	5 min/ biweekly/ 12 weeks	-Group music listening with rhythmic exercise significantly increased cognitive function and life satisfaction, in addition decreased depression and anxiety in Korean nursing home residents
				Control	20	3,17	82.6 ± 9.3	Standard care	12 weeks	
Kwok et al. (2011) (24)	Hong Kong	Non-RCT	Mild Cognitive Impairment or Dementia	Music	15	2,22	77.63 ± 5.91	Listening, singing	3 times a week/ not specified	-Continuous cognitive training with memory-training was shown to have less benefit than memory-training alone in older people.
				Control	9		78.33 ± 5.59	Standard care	Not specified	
Li et al. (2015) (25)	Taiwan	Non-RCT	Mild Dementia	Music	52	5,15	76.7 ± 8.45	Listening	30 min/daily/ 6 months	-Classical music listening therapy showed no additional benefits on global cognition and daily function in mild AD patients for 6 months. -Listening therapy confirms cognition effect on abstraction and short-term memory
				Control	35	8,13	80.8 ± 5.94	Standard care	6 months	
Li et al. (2016) (18)	China	RCT	Mild–Severe Dementia	Music	19	7,12	83.1 ± 4.1	Singing	40–50 min /16 weeks	-Chinese folk recreational program – involving art, game, and music – significantly improved MMSE scores in older people with dementia. -Folk recreation program can improve cognitive function, ability of daily living and reduce behavioral and psychological symptoms.
				Control	21	5,16	81.8 ± 6.7	Standard care	16 weeks	
Lyu et al. (2018) (26)	China	RCT	Mild Dementia	Music	97	40,57	68.9 ± 7.1	Singing	30–40 min/ twice daily/ 3 months	-Music therapy enhanced memory and language ability in patients with mild AD. -Music therapy reduced psychiatric symptoms of patients with advanced AD and the level of the distress encountered by their caregivers. -Training sessions of singing songs are more effective than reading lyrics of the songs
				Control	95	39,57	69.9 ± 7.9	Standard care	3 months	
Parlak et al. (2023) (27)	Turkey	Non-RCT	Mild–Moderate Dementia	Music	10	4,6	78.40 ± 7.93	Listening, singing	5–10 min/ once a week/ 6 weeks	-Holistic therapy – using music, reminiscence, and reality orientation – showed significant improvements in cognition, depression level, and quality of life in people with AD.
				Control	10	5,5	70.90 ± 6.49	Standard care	6 weeks	
Perez-Ros et al. (2019) (28)	Spain	RCT	Mild Dementia	Music	47	16,31	80.06 ± 7.63	Listening, singing, movement	60 min/ 5 times a week/ 8 weeks	-Group intervention of listening to preferred music improved functional condition and mood state, while cognitive function did not worsen in patients with cognitive impairment
				Control	72	42,30	80.80 ± 7.36	Occupational therapy	8 weeks	
Sun et al. (2021) (29)	Taiwan	Non-RCT	Mild Cognitive Impairment	Music	62	7,55	74.29 ± 7.53	Listening, movement	120 min/ once a week/ 12 weeks	-Music therapy with physical activity improved frailty, physical fitness, cognitive function, and depression among the community older adults
				Control	60	20,40	73.50 ± 6.14	Standard care	12 weeks	
Tang et al. (2018) (30)	China	RCT	Mild–Moderate Dementia	Music	38	21,17	76.39 ± 4.86	Listening, singing, playing	50 min/ 3 times a week/ 12 weeks	-Music therapy effectively maintained cognitive function in older residents with dementia, alleviated the symptoms of apathy, and improved verbal communication ability
				Control	39	18,21	75.38 ± 4.94	Standard care	12 weeks	
Wang et al. (2018) (31)	China	RCT	Mild Dementia	Music	30	10,20	70.4 ± 7.5	Listening, singing	30–50 min/ 3 times a day/ 3 months	-Music therapy combined with pharmacological treatment led to greater improvements in the cognitive function and mental behavior of AD patients
				Control	30	12,18	69.1 ± 7.2	Standard care	3 months	

Methodological quality was evaluated through systematic risk of bias assessment (Appendices A, B). Studies implementing intention-to-treat (ITT) protocols demonstrated consistently low bias risk across assessment domains, with notable exceptions in outcome reporting where most investigations lacked explicit confirmation of adherence to pre-registered analytical frameworks established prior to data unblinding. Kim warranted particular concern given authors’ explicit acknowledgment of potential measurement bias in outcome assessment (23). Per-protocol analyses revealed moderate concerns stemming from participant non-adherence to assigned interventions and inadequate specification of analytical plan adherence. Non-randomized studies achieved Newcastle-Ottawa Scale scores ≥7 (Appendix C), indicating acceptable methodological rigor across cohort selection, comparability, and outcome assessment domains. Both raters assigned identical risk of bias scores for all items, indicating perfect agreement.

**Figure 2:**
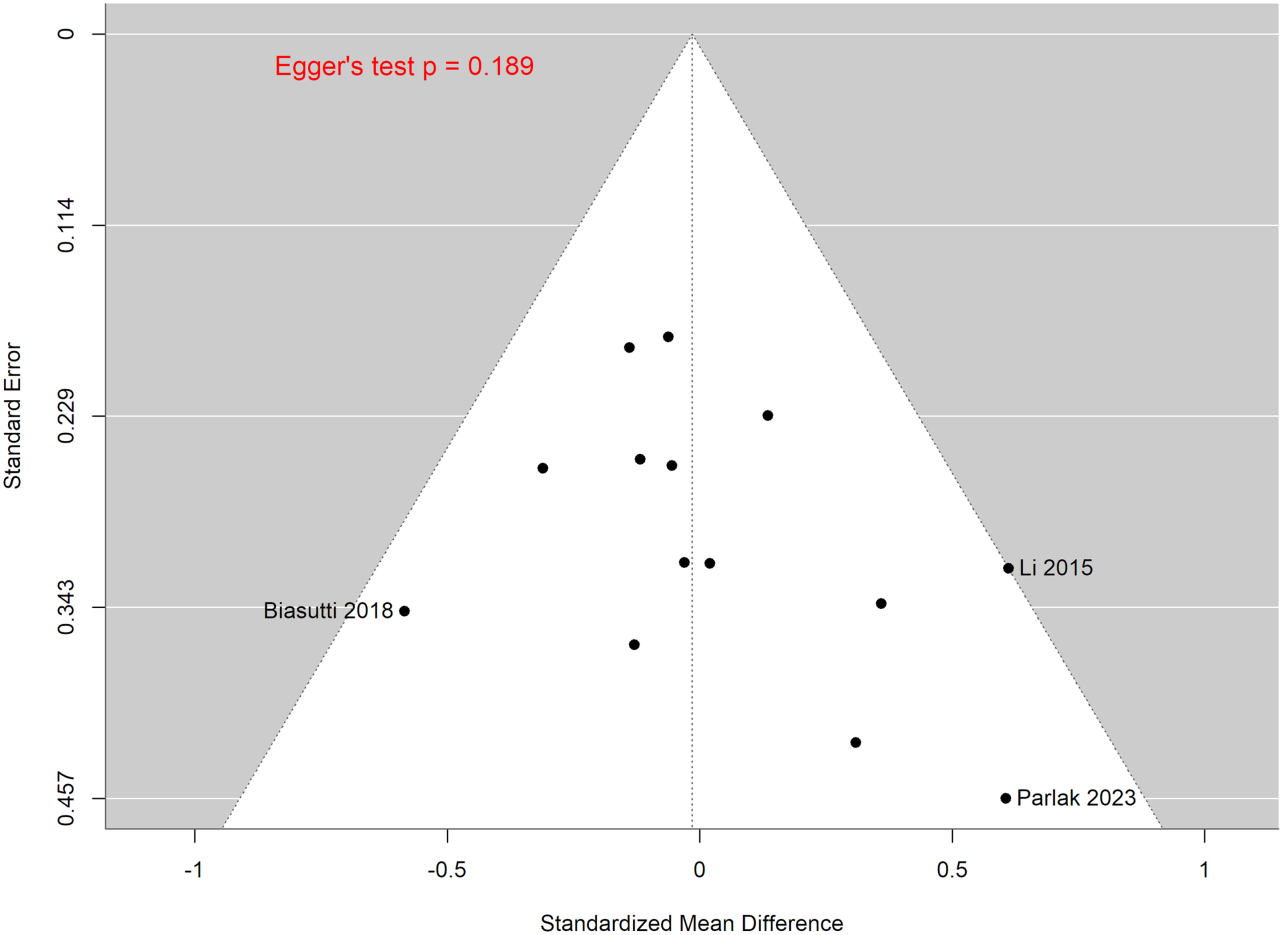
Funnel plot assessing small-study effects and publication bias.

To further assess the bias risk, a funnel plot of the 14 included studies was generated (Figure 2). The resulting plot suggested moderate asymmetry, particularly among smaller studies such as Biasutti and Mangiacotti (19), Li et al. (18), and Parlak et al. (27). In contrast, the larger studies were shown to be more symmetrically distributed around the pooled estimate. However, Egger’s regression test showed no statistical significance to the asymmetry (*p* = 0.189), indicating no strong evidence of publication bias influencing the pooled estimates. In addition, sensitivity analysis using a leave-one-out approach (Appendix E) showed that exclusion of any individual study did not substantially affect the pooled effect size. All the recalculated estimates remained close to the overall pooled effect, indicating validity of the previous bias risk findings.

**Figure 3:**
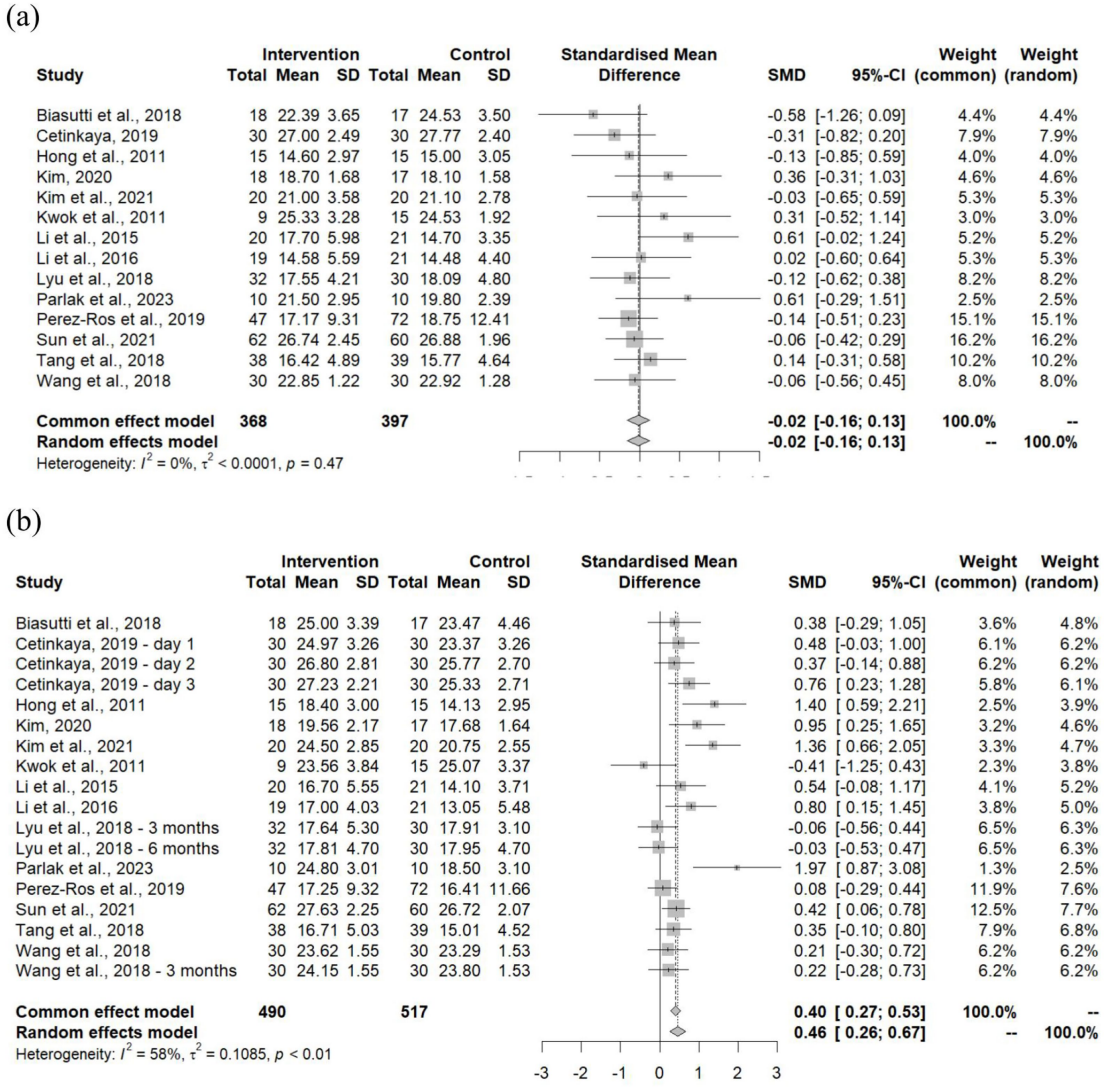
Forest plot of MMSE outcome at **(a)** baseline and **(b)** follow-up.

The MMSE scores of all selected studies at baseline and follow-up were compared and analyzed to generate forest plots (Figures 3a,b). The baseline forest plot showed a leftward shift with a standardized mean difference (SMD) of −0.02, showing no significant difference (95% CI: −0.16–0.13) between groups, which is further supported by the low heterogeneity (*I*^2^ = 0%, *p* = 0.47). This indicates that the baseline MMSE scores throughout all studies were highly consistent. In contrast, the follow-up forest plot showed a rightward shift with an SMD of 0.46 (95% CI: 0.26–0.67), indicating a moderate improvement in cognitive function from the intervention, though some variability was present between studies (*I*^2^ = 58%, *p* < 0.01).

### 3.1 Subgroup analyses

Multiple subgroup analyses were performed to examine effects across study design, trial type, treatment approach, study duration, and publication year.

### 3.2 Study design

**Figure 4:**
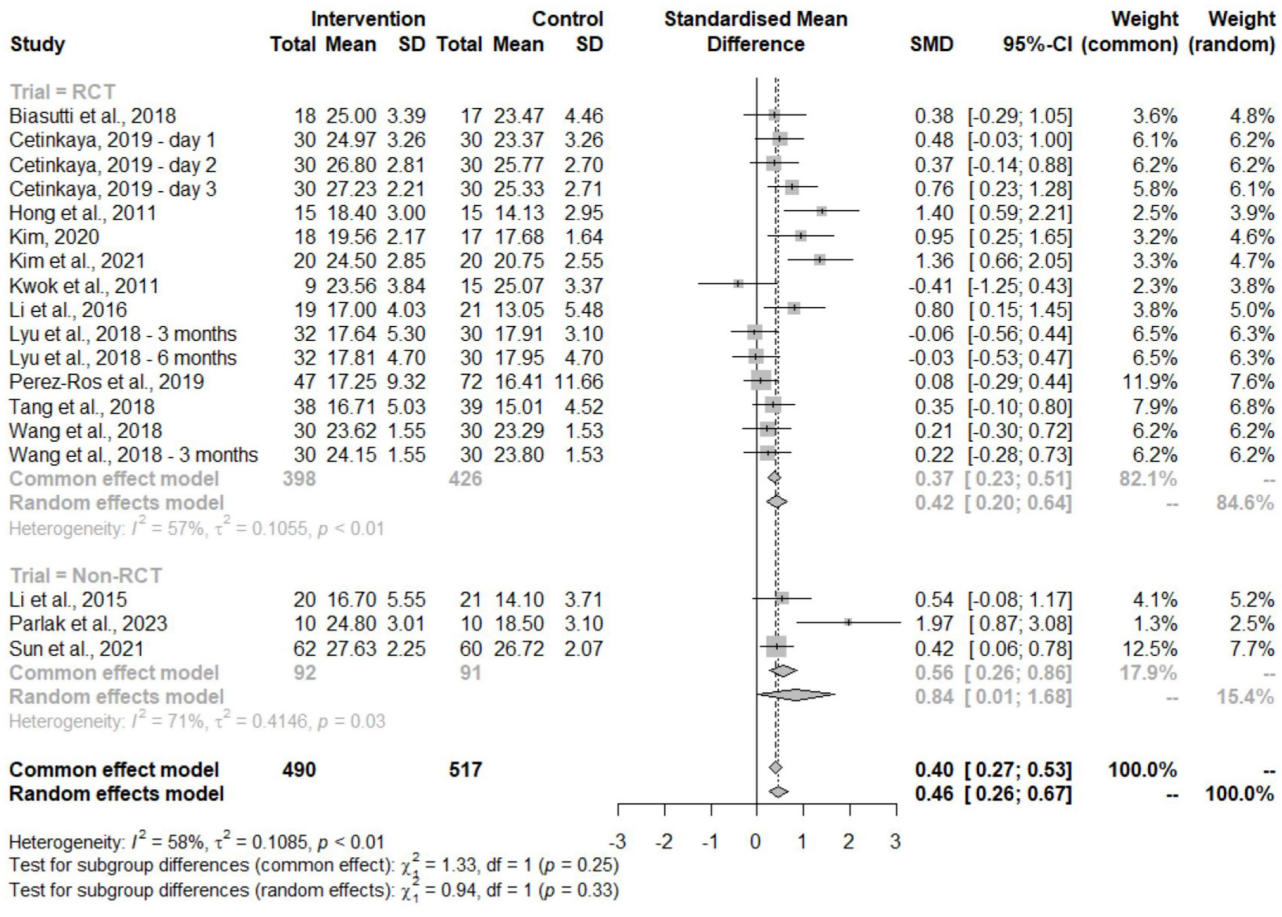
Forest plot of MMSE outcome based on trial type.

Studies were classified as randomized controlled trials (RCTs) or non-RCTs to evaluate methodological quality (Figure 4). The pooled analysis showed a statistically significant positive effect (SMD = 0.46, 95% CI: 0.26–0.67) with moderate heterogeneity (*I*^2^ = 58%, *p* < 0.01). Both study types produced consistent results with no significant subgroup differences (*χ*^2^ = 0.94, df = 1, *p* = 0.33). RCTs demonstrated moderate effects (SMD = 0.42, 95% CI: 0.20–0.64), while non-RCTs showed larger effect sizes with wider confidence intervals (SMD = 0.84, 95% CI: 0.01–1.68), indicating greater variability in non-randomized designs.

### 3.3 Treatment approach

**Figure 5:**
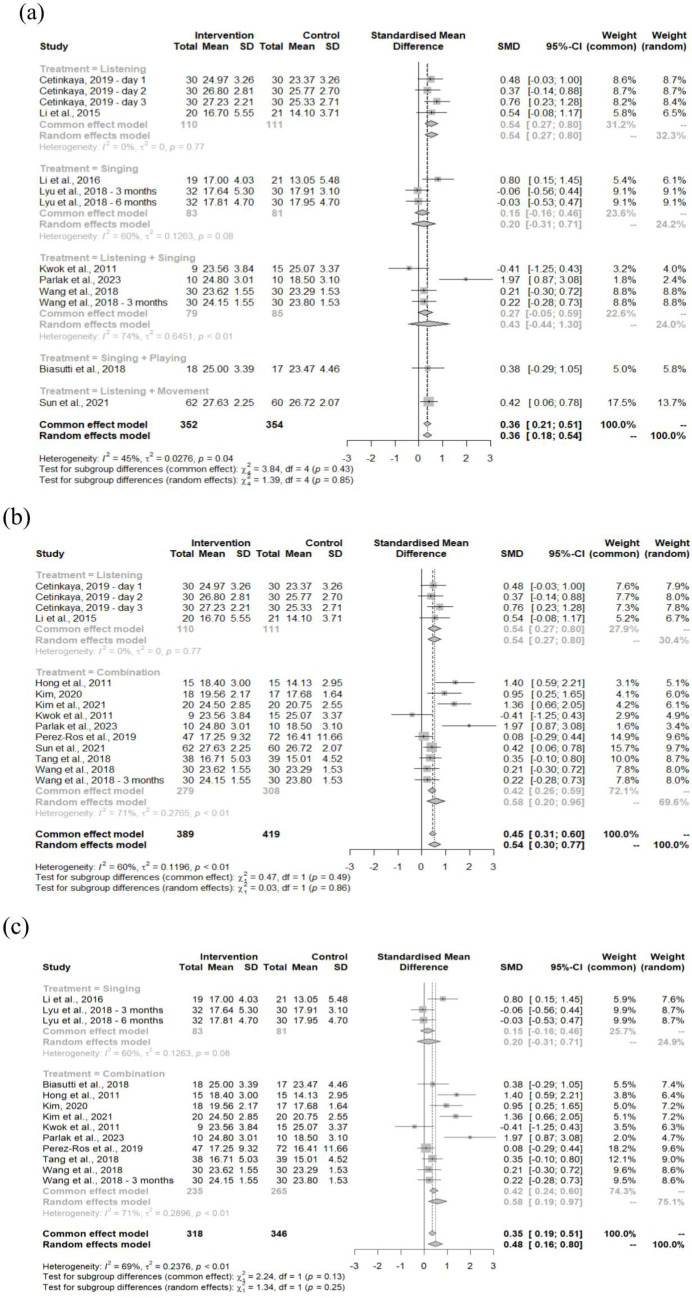
Forest plot of MMSE outcome based on **(a)** treatments, **(b)** listening vs. combination, and **(c)** singing vs. combination.

Three treatment comparisons were analyzed: overall combined treatment (Figure 5a), listening versus combination (Figure 5b), and singing versus combination (Figure 5c).

### 3.4 Overall treatment

Analysis showed moderate, statistically significant improvement (SMD = 0.36, 95% CI: 0.18–0.54) with low heterogeneity (*I*^2^ = 45%, *p* = 0.04) and no significant between-group differences (*χ*^2^ = 1.39, df = 4, *p* = 0.85). Listening interventions produced the largest effects (SMD = 0.54, 95% CI: 0.27–0.80). Comparing listening alone to combination approaches (incorporating singing, playing, or movement), both demonstrated significant positive effects (SMD = 0.54, 95% CI: 0.30–0.77). Combination treatments showed stronger effects (SMD = 0.58, 95% CI: 0.20–0.96) than Listening-only interventions (SMD = 0.54, 95% CI: 0.27–0.80), though differences were not significant (*χ*^2^ = 0.03, df = 1, *p* = 0.86) with moderate heterogeneity (*I*^2^ = 60%, *p* < 0.01). Singing versus combination comparisons revealed smaller but significant overall effects (SMD = 0.48, 95% CI: 0.16–0.80). Aligned with the listening vs. combination comparison, singing interventions alone produced minimal, non-significant improvements (SMD = 0.20, 95% CI: −0.31–0.71), while combination approaches including singing showed significant benefits (SMD = 0.58, 95% CI: 0.19–0.97). This contrast produced substantial heterogeneity (*I*^2^ = 69%, *p* < 0.01), though subgroup differences remained non-significant (*χ*^2^ = 1.34, df = 1, *p* = 0.25).

### 3.5 Treatment duration

**Figure 6:**
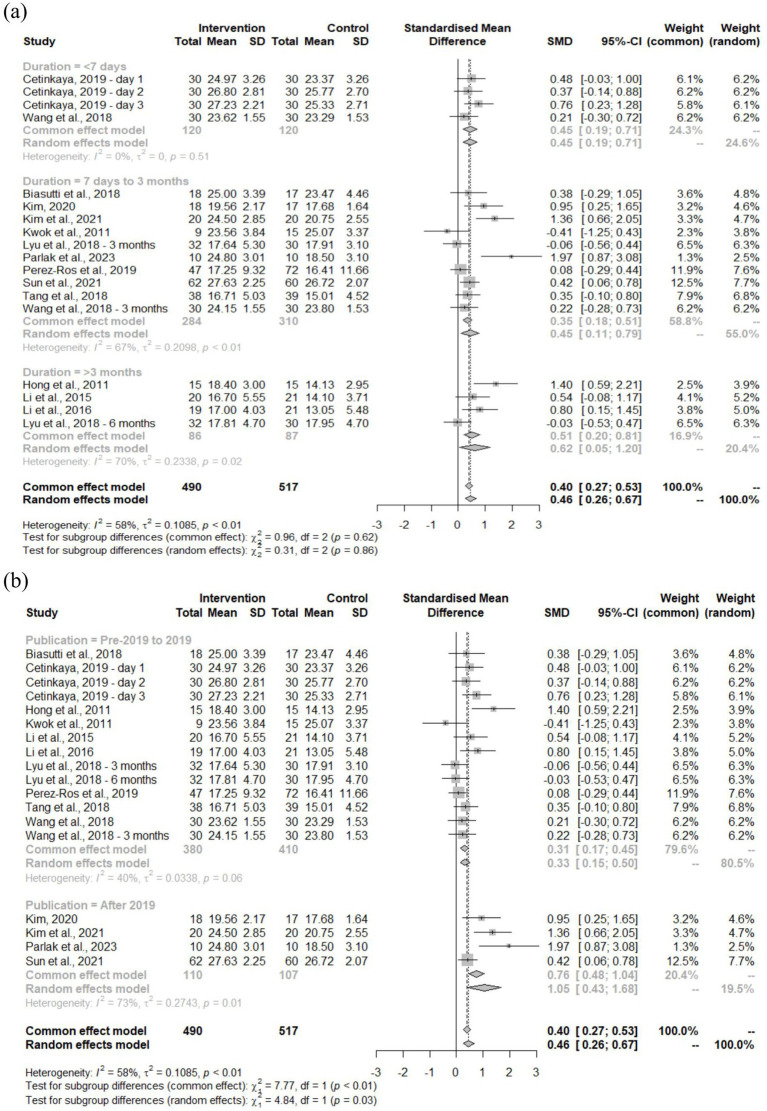
Forest plot of MMSE outcome based on **(a)** treatment duration and **(b)** publication year.

Studies were categorized by treatment duration: <7 days, 7 days to 3 months, and >3 months (Figure 6a). The largest improvements occurred with treatments lasting >3 months (SMD = 0.62, 95% CI: 0.05–1.20), followed by <7 days (SMD = 0.45, 95% CI: 0.19–0.71) and 7 days to 3 months (SMD = 0.45, 95% CI: 0.11–0.79). Despite moderate heterogeneity across studies (*I*^2^ = 58%, *p* < 0.01), subgroup differences were not significant (*χ*^2^ = 0.31, df = 2, *p* = 0.86).

### 3.6 Publication year

Analysis comparing studies published before versus after 2019 revealed significant subgroup differences with an SMD of 0.46 and 95% CI of 0.26–0.67 (Figure 6b; *χ*^2^ = 4.84, df = 1, *p* = 0.03). Post-2019 publications demonstrated substantially larger effects (SMD = 1.05, 95% CI: 0.43–1.68) compared to earlier publications (SMD = 0.33, 95% CI: 0.15–0.50). Heterogeneity remained moderate at *I*^2^ = 58% (*p* < 0.01).

## 4 Discussion

The goal of this meta-analysis study is to evaluate the impact of music therapy on neuropsychological functions. This study revealed that music therapy interventions were associated with overall improvements in global cognitive function, as measured by the MMSE scores.

By searching through the PubMed, Embase, and Cochrane Library databases, 14 studies published between 2010 and 2025 were selected to be analyzed. Most of the study participants were older adults with varying conditions such as mild cognitive impairment, dementia, and hip or knee surgery receiving music therapy through a variety of exposure methods. Before interpreting the findings from selected studies, a key consideration was the quality and reliability of each study result; thus the risk of bias assessment was conducted with the RoB-2 tool and NOS.

Most of the selected studies demonstrated low to moderate risk of bias. The RCTs with an ITT approach generally had low risk of bias, though most failed to specify whether outcome reporting aligned with a pre-established analysis plan; thus raising the possibility of selective reporting bias by this omission. On the other hand, the PP studies showed some concerns in their bias risk with most studies having participants who were non-adherent to the assigned interventions and incomplete transparency when reporting outcomes. In addition, there were non-randomized studies which were assessed based on NOS. Results reveal a relatively strong methodological quality from these studies, with all studies scoring 7 or higher. An additional evaluation by creating a funnel plot showed moderate asymmetry which may indicate potential publication bias or small-study effects – mainly due to the tendency of smaller studies to report more favorable outcomes. However, with a relatively small study number (*n* = 14) in this meta-analysis, visual interpretation alone may not be sufficient; hence the inclusion of an additional sensitivity analysis for further validation. Contrasting the funnel plot, the sensitivity analysis indicated robustness in overall findings, as no single study showed any disproportionate influence on the pooled effect size; suggesting that the observed effect of music therapy on MMSE scores to be consistent across studies. Taken together, bias risks of the included studies are validated to be moderately low, with some risk potentially arising from small-study effects (32).

With the quality and reliability of the studies being validated, the MMSE results were then analyzed and compared between studies. At baseline, the MMSE scores were shown to be consistent between both intervention and control groups across all studies, confirming comparability of the cognitive profiles at the starting point of the studies; thus providing further support to the validity of the outcomes that will be observed. The follow-up analysis revealed moderate improvement in the MMSE scores with statistical significance (SMD = 0.46, 95% CI: 0.26–0.67). This suggests that music therapy may effectively enhance cognitive function in older adults with various conditions. Such finding is in line with results from a prior meta-analysis by Bian et al. (15), wherein an improvement in MMSE score of the music therapy group (SMD = 0.86, 95% CI: 0.07–1.66, *p* = 0.03) in comparison to the control group was found; thus supporting the findings in this study. It is notable that this study included a bigger sample size with more recent studies, hence the difference in SMD which may suggest a more moderate yet consistent effect. Similarly, a meta-analysis by Lin et al. (14) found improvements in NPI scores which highlight the cognitive improvements of participants in the intervention group compared to their control counterparts. While the NPI evaluation primarily captures the behavioral disturbances in dementia, some studies have been found to use cognitive (i.e., MMSE) and behavioral (i.e., NPI) scales complementarily; hence improvements in NPI scores may potentially reflect underlying cognitive benefits that further substantiate findings of the present study (33).

The improvement in cognitive function shown in participants treated with music therapy may be accounted for by its ability to promote neuroplasticity in cognitive domains vulnerable to age-related deterioration. Although the studies included in this meta-analysis did not directly investigate the neurobiological mechanisms of music therapy, converging evidence from neuroimaging and electrophysiological studies provides possible explanations for the observed cognitive improvements. Neuroimaging studies have shown enhanced connectivity in the prefrontal cortex and default mode network – regions which have been largely implicated in executive control and memory (32, 33). Beyond connectivity, structural magnetic resonance imaging (MRI) evidence indicates that music-based interventions can increase gray matter volume in the frontal and temporal regions of patients with stroke and traumatic brain injury, subsequently showing improvements in executive function (34). This structural evidence is complemented by electrophysiological findings in a RCT by Ahuja et al. (35) which found enhanced P300 amplitudes at frontal and central sites following music listening in patients with schizophrenia, potentially linking this intervention with improved attention and working memory. Importantly, in the context of dementia, Jacobsen et al. (12) demonstrated that regions associated with musical memory, such as the caudal anterior cingulate and ventral pre-supplementary motor area, remain relatively preserved in Alzheimer’s disease. This finding suggests that music-based interventions may be particularly effective in engaging these networks to support cognitive and quality of life improvements. Collectively, these findings are consistent with studies which have hinted that music therapy may exert its therapeutic effect via dopaminergic pathways, activating the reward circuits that facilitate learning and memory (36, 37). Aside from cognitive improvements, music therapy has also been shown to enhance mood and motivation in patients; thus indirectly influencing cognitive function by creating a more conducive environment for improvements (10). Further supporting these findings is a study by Blood and Zatorre (38) which used positron emission tomography (PET) to measure changes in the regional cerebral blood flow of patients when listening to a music of choice, showing that music-evoked pleasure changed cerebral blood flow in key brain regions involved in pleasure and reward-seeking (e.g., ventral striatum, amygdala, ventral medial prefrontal cortex). The dopaminergic system related to this reward circuitry projects to the nucleus accumbens (NAc) of the ventral striatum, thus triggering dopamine release.

To further explore the potential sources of heterogeneity and assess the consistency of music therapy efficacy across different conditions, several subgroups were analyzed. Given the variation in study designs, patient conditions, intervention methods, and treatment durations among the selected studies, the subgroup analyses would provide a structured approach to examine whether these factors contribute to the observed improvement. Furthermore, temporal subgrouping of studies published pre- and post-2019 was included to investigate whether recent developments in clinical research or therapeutic practice may have an influence on the reported effect sizes. Hence the subgroup analysis was planned to enhance the interpretability and applicability of the findings across various clinical and research settings.

In the study design subgroup analysis, it was found that the efficacy of music therapy on cognitive benefits was not only limited to RCTs. Non-RCTs were able to demonstrate similar effect sizes, though the confidence intervals were wider with higher heterogeneity. This reflects the higher risk of variability in design or patient characteristics in non-RCT studies. It has been observed that non-RCT studies have consistently increased heterogeneity and holds more weight to overall estimates in meta-analyses due to larger sample sizes, especially when pooled with RCTs. A few ways to mitigate this is by prioritizing non-RCT studies with lower risk of bias and applying statistical approaches designed to account for potential bias. These strategies, together with the accumulation of larger and more rigorous RCTs, may help refine effect size estimates and reduce heterogeneity in future syntheses (39).

To analyze the music therapy treatment or intervention methods, the studies were further grouped into general, listening-based, and singing-based music therapy. Listening-based therapy was mainly carried out individually by the subjects with assistance from a therapist or caretaker, while singing-based therapy was primarily done in group settings. A key consideration in interpreting the following findings is the substantial heterogeneity in intervention methods across included studies. Particularly, each method may recruit different brain regions; listening-based therapy would predominantly activate auditory and limbic pathways linked to emotion and reward, while the more active or multimodal methods such as singing recruits the auditory and motor brain regions in addition to regions associated with working memory (40–42). In addition, musical preference was only explicitly implemented in two studies (27, 28) while the remaining trials employed standardized classical or familiar folk or trot music. This may be an additional factor for heterogeneity as the sense of anticipation that is triggered by the familiarity to the music has been reported to cause dopamine release (43). The subgroup analysis showed that listening-based music therapy had slightly better outcomes than its combination or singing-based approaches. This suggests that passive engagement through listening-based music therapy may be particularly effective. Notably, the singing-based therapy by itself yielded the least effect and was not statistically significant, but combination with other methods showed some improvement. This finding aligns with a study by Tsoi et al. (44), which revealed that music therapy has been demonstrated to decrease anxiety, agitation, and behavioral problems – particularly in people with dementia. One possible explanation for the better cognitive outcomes in listening-based music therapy compared to singing-based may lie in the differential neural resource allocation and auditory network plasticity. Receptive music listening primarily activates early auditory and limbic circuits, enhancing sensory control and reward-related dopaminergic signaling without the higher cognitive demand of motor planning and coordination. Schneider et al. (45) demonstrated that short-term passive listening therapy can induce measurable neuroplastic changes in the auditory cortex tonotopy and temporal processing, resulting in improved auditory discrimination and attention modulation. The observed rapid adaptations suggest that listening-based therapy may facilitate low-level auditory pathway refinement and top-down attentional tuning, thus leading to more efficient sensory integration. In contrast, active music making requires multimodal and executive networks, which may cause the need to distribute cognitive resources and attenuate focused auditory processing. Nonetheless, a RCT by Särkämö et al. (13) observed that listening- and singing-based therapy both have beneficial outcomes on patients with early dementia; listening-based music therapy induced long-term quality of life improvements, while singing-based music therapy immediately improved short-term and working memory tasks post-intervention. These findings align with the mechanisms proposed in the current meta-analysis, particularly the enhancement of auditory connectivity, attentional control, and sensory efficiency; hence providing a biological hypothesis for the observed clinical benefits of receptive music-based interventions. Listening-based music therapy may also provide a more accessible therapeutic option due to its simplicity and convenience in application at different environmental settings (e.g., nursing homes). In addition, the potential reluctance of older adults with conditions such as depression, dementia, and other illnesses or physical disabilities presents listening-based music therapy as a more adaptable option.

After analyzing the treatment method, duration emerged as another potential factor to be analyzed. Notably, the results show an interesting finding that the short-term intervention duration of >3 months yielded the biggest improvement. This may indicate that continuous or long-term music therapy may be more beneficial in improving cognitive function, a finding supported by the results in a study by Lyu et al. (26) wherein patients with Alzheimer’s disease showed the highest improvement when treated with music therapy for 3 months. This pattern of long-term music therapy benefit may be accounted for by the brain neuroplasticity, which is the ability to undergo modifications of functional or structural nature – ranging from cellular to tissue level – as a response to experience or injury. These changes may manifest through the neuron morphology, synaptic weight changes, pruning, or cortical remapping (46–48). This has been investigated in multiple studies which observed that corpus callosum size is positively correlated with the duration of musical training, as reflected in the substantially larger corpus callosum in musicians compared to non-musicians (49–51).

A particularly noteworthy finding within the subgroup analyses is the marked increase in effect size within studies published post-2019. With the onset of the coronavirus disease 2019 (COVID-19) pandemic shortly after 2019, the relevance of accessible, non-pharmacological interventions like music therapy has increased dramatically (52). This may be a plausible catalyst to the advancement in intervention design or increase in academic rigor, with more studies employing standardized cognitive assessments, well-defined intervention protocols, and more representative samples. Moreover, music therapy design may have been more refined through the increased use of digital platforms – effectively enhancing participant engagement and neural responsiveness. Collectively, these developments likely contributed to the higher efficacy observed in post-2019 studies, reflecting both advances in experimental quality and the psychosocial changes brought about by the pandemic era.

Music therapy offers a cost-effective, non-invasive intervention that is easily applicable and can be integrated seamlessly into existing care routines for patients of different conditions. The ability of music therapy to drive cognitive function improvements in addition to reducing neuropsychiatric symptoms makes it an invaluable adjunct to pharmacological treatments (53, 54). Moreover, the accessibility and low risk of adverse effects of music therapy further support its role as an ideal intervention for various neurodegenerative and non-neurodegenerative conditions.

Despite the promising findings of this study, there are several limitations that should be considered. First, some heterogeneity was observed across studies, which may cause some challenge in making direct comparisons and generalizability. Second, it is noteworthy that several of the included studies had relatively small sample sizes, potentially affecting the reliability and statistical power of the results. Third, despite efforts to only include studies with robust methodological quality, publication bias and selective reporting may potentially still be of concern. Furthermore, with most studies lacking long-term follow-up durations, it may be difficult to assess the sustainability of the cognitive improvements over time. Presently, the exact mechanisms underlying cognitive enhancement through MT remain underexplored. While several neuroimaging and electrophysiological studies have suggested involvement of the dopaminergic pathway and enhanced connectivity in the default mode network, these findings remain inconclusive across the general diverse populations.

Therefore, future research should prioritize the standardization of intervention protocols with clearly defined auditory stimuli and consistent outcome measures, longitudinal and larger sample studies to assess durability of cognitive improvements, and mechanistic studies using neuroimaging or electrophysiological tools to elucidate the mechanisms underlying the influence of music therapy on brain plasticity and cognition. Additionally, efforts should focus on clinical translation to integrate music therapy into rehabilitative and preventive healthcare systems, particularly for aging populations with limited mobility.

## 5 Conclusion

This meta-analysis demonstrates that auditory-based music therapy significantly improves cognitive function as measured by MMSE scores, with long-term listening interventions (>3 months) showing particular efficacy. The passive nature of auditory stimulation offers an accessible, non-invasive treatment option for older adults with physical or cognitive limitations, making it readily implementable in clinical settings. While these findings support integrating music therapy into clinical practice for cognitive decline, the heterogeneity of current protocols highlights the need for standardization and further investigation of underlying neurobiological mechanisms to optimize therapeutic applications across diverse patient populations.

## Data Availability

The original contributions presented in the study are included in the article/Supplementary material, further inquiries can be directed to the corresponding authors.
