# Supplementary material for: Cognitive enhancement through music therapy: meta-analytic evidence across clinical population
Source: Front Public Health. 2026 Jan 8;13:1735470. doi: 10.3389/fpubh.2025.1735470 (PMC12823501; doi:10.3389/fpubh.2025.1735470)

**Appendix A.** Risk of bias analysis on intention-to-treat (ITT) studies


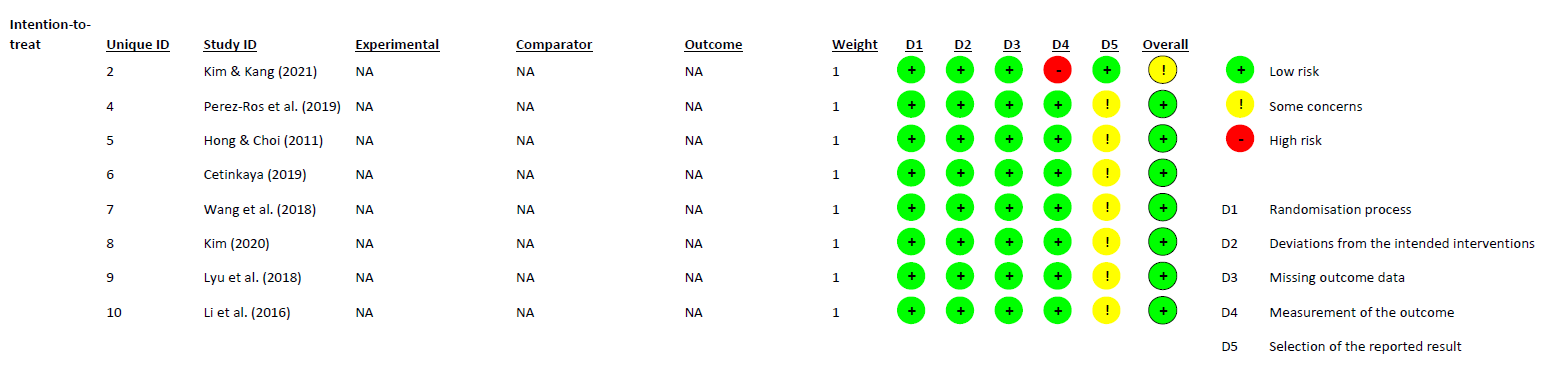


**Appendix B.** Risk of bias analysis on per protocol (PP) studies


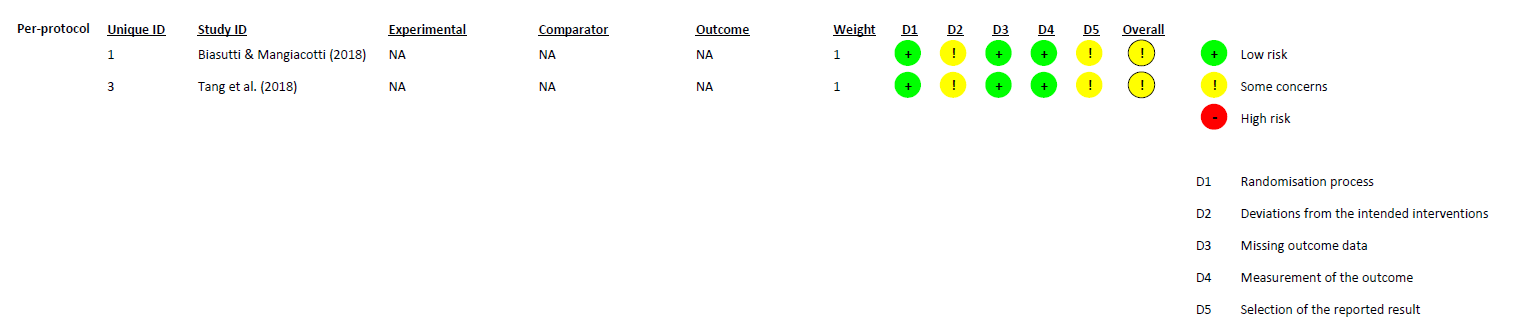


**Appendix C. Newcastle-Ottawa Scale (NOS) Score of Non-Randomized Studies.**

| First Author, Year | Selection | Comparability | Outcome | NOS |
| --- | --- | --- | --- | --- |
| Kwok et al., 2011 | 4 | 2 | 3 | 9 |
| Li et al., 2015 | 3 | 2 | 2 | 7 |
| Parlak et al., 2023 | 3 | 2 | 3 | 8 |
| Sun et al., 2021 | 4 | 2 | 3 | 9 |

**Appendix D.** Leave-One-Out Sensitivity Analysis of Included Studies


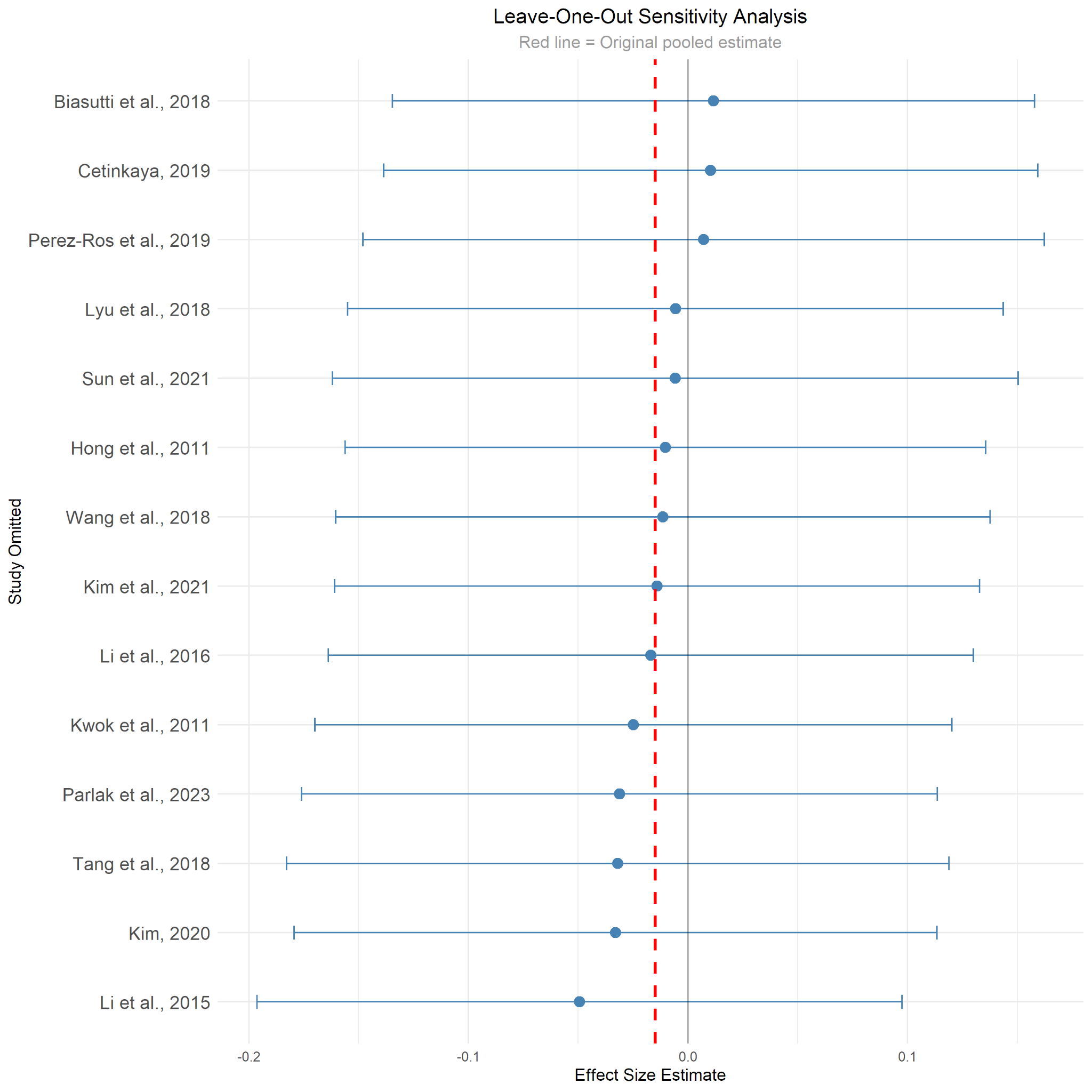

Supplement: Supplementary file 1 [file Table_1.DOCX]
